# Whole-Genome-Sequencing Analysis of the Pathogen Causing Spotting Disease and Molecular Response in the *Strongylocentrotus intermedius*

**DOI:** 10.3390/microorganisms13092019

**Published:** 2025-08-29

**Authors:** Shufeng Li, Fenglin Tian, Yongjie Wang, Haoran Xiao, Zijie Zhou, Lina Cao, Lingshu Han, Junxiao Sun, Chong Zhao, Jun Ding

**Affiliations:** 1 Liaoning Provincial Key Laboratory of Northern Aquatic Germplasm Resources and Genetics and Breeding, Dalian Ocean University, Dalian 116023, China; 2 Key Laboratory of Mariculture & Stock Enhancement in North China’s Sea, Ministry of Agriculture and Rural Affairs, Dalian Ocean University, Dalian 116023, China

**Keywords:** *Strongylocentrotus intermedius*, spotting disease, *Vibrio splendidus*, 16S rDNA, whole-genome sequencing, RNA-seq

## Abstract

Sea urchin aquaculture has experienced remarkable growth in recent years. However, this growth has been accompanied by increased disease prevalence. Notably, spotting disease has particularly severe impacts. In this study, we isolated the pathogen HZ-3-2 from 10 sea urchins with spotting disease, and it was identified as *Vibrio splendidus* through morphological observations, 16S rDNA sequencing, and whole-genome sequencing. Subsequently, experimental infection confirmed that *V. splendidus* (HZ-3-2) is the causative agent of spotting disease in this outbreak. The drug sensitivity confirmed the presence of drug resistance genes, such as *CPR*, *QNRS5*, and *rsmA*, which were identified in the genome. The tests indicated that *V. splendidus* was sensitive to various antibiotics, including fluoroquinolones and florfenicol. Finally, we used the transcriptome to explore the molecular response of the diseased sea urchin. Compared to the control group, a group of sea urchins immersed in a pathogen suspension with a concentration of 10^7^ CFU/mL (group M) resulted in 439 annotated differentially expressed genes. KEGG pathway analysis indicated significant activation of cholesterol metabolism and starch and sucrose metabolism in the *S. intermedius*. This study highlights the genes *NPC1*, *AMY2A*, and *MGAM* as critical regulators of energy metabolism, and cholesterol synthesis in infected sea urchins. These findings confirm *V. splendidus* as the bacterium responsible for spotting disease and provide valuable insights into the intestinal molecular response of *S. intermedius* to infection.

## 1. Introduction

The global aquaculture industry has proliferated during the last decades, particularly with the increased use of high-density farming techniques. This expansion has led to disease outbreaks in aquaculture animals, displaying explosive and cyclical epidemic patterns [1]. Bacterial diseases occur frequently and pose important challenges to the growth of the global sea urchin aquaculture [2,3]. In China, sea urchin production reached 470.6 tons in 2023, covering an aquaculture area of 16,800 hectares [4]. Among these, *Strongylocentrotus intermedius* is a primary farmed species in northern China. Currently, its annual production exceeds 200 tons [5] and yields at least 18 million freshly metamorphosed juveniles, which can be used to initiate a culture annually [5]. Sea urchins are also eaten or farmed in Norway [6], the United States [7,8], and Russia [9,10]. The rational development and utilisation of sea urchin resources positively protect the marine ecological balance [11]. However, with the development of aquaculture, outbreaks of spotting disease have become more frequent. This impacts the development of China’s sea urchin aquaculture [12].

The clinical signs of spotting disease include the appearance of red or purplish-red spots or patches on the test of sea urchins, ulceration and tissue damage at the site of infection, and spine shedding. Eventually, the sea urchin’s internal contents, such as gonadal tissue leak due to test fragmentation, ultimately resulting in the sea urchin’s death [12,13]. During the summers of 2002 and 2003, large-scale mortalities of *S. intermedius* farmed along the coast of Dalian occurred due to outbreaks of spotting disease, resulting in substantial economic losses for local aquaculture farmers. The identification of pathogens was a prerequisite for the prevention and control of aquatic diseases. Previous studies have identified that the pathogens responsible for causing spotting disease are mainly *Vibrio* sp. [12,14], *Flexibacter* sp. [15], and *Enacibaculum* sp. [16]. In 2005, an outbreak of spotting disease caused by other species of *Vibrio* was reported [17]. However, it is unclear which *Vibrio* species caused the disease in 2005. This undoubtedly poses a severe challenge to using immunological methods for preventing and controlling “spotting disease” in sea urchin aquaculture. Therefore, it is crucial to isolate and identify new bacteria associated with spotting disease.

In aquaculture, various methods for disease prevention and control have been identified. For instance, probiotics such as *Clostridium butyricum* [17] and *Bacillus* [18] were added during the farming process to enhance animal immunity [19]. Alternatively, chemical methods such as sodium hypochlorite and ozone were used for disinfection [20]. These measures aimed to reduce the occurrence of diseases during the farming process, thereby improving the survival rate of aquatic animals. However, in the actual production processes of aquaculture, the use of antibiotics to address bacterial diseases remains one of the most cost-effective and efficient methods [21]. Conducting drug sensitivity tests on an isolated bacterium is essential for identifying effective antibiotics. Therefore, recommending appropriate antibiotics based on relevant aquaculture medication standards is constructive for preventing and controlling the spotting disease of sea urchins.

Gene expression analysis serves as a link between genetic material and traits, and it can be regulated by changes in the external environment to adapt to environmental variations [22]. Transcriptome sequencing remains one of the effective methods for studying gene regulatory expression and analyzing biological processes at the molecular level [23,24]. In recent years, transcriptomics has been applied to study the immune response mechanisms of *S. intermedius* in response to the infection of *Vibrio harveyi* [1], as well as to elucidate the role of phagocytosis as the primary immune response in sea urchins [25]. These experiments primarily focused on the molecular response of the coelomic fluid of sea urchins to the pathogen. As an organ in aquatic animals, the intestine also has certain immune functions [26]. However, it remains unknown whether the intestine of sea urchins possesses similar immune functions and molecular response changes. In summary, this study employed transcriptome sequencing to investigate the molecular response mechanisms of the intestine in *S. intermedius* following spotting disease infection, aiming to provide reasonable hypotheses and insights.

In this study, we isolated and identified the pathogen (HZ-3-2) of spotting disease. Following this, we assess the drug sensitivity of the HZ-3-2 strain and analyze the molecular response of *S. intermedius* following infection with HZ-3-2. This study provides theoretical references for preventing and controlling the spotting disease of *S. intermedius*.

## 2. Materials and Methods

### 2.1. Test Animals

The sea urchins (*S. intermedius*) used in this experiment were 1–1.5 years old and cultivated at an aquaculture facility in Lvshun, Dalian, China. We randomly selected 100 healthy sea urchins with a mean test diameter of 40.27 ± 4.53 mm, test height of 20.63 ± 2.9 mm, and body weight of 25.98 ± 6.55 g. They were transported to the Key Laboratory of Mariculture & Stock Enhancement in North China’s Sea, Ministry of Agriculture and Rural Affairs, Dalian Ocean University, on 1 March 2024. The temperature was increased by 0.5 °C per day until it reached a constant value of 22 ± 0.5 °C, and the water temperature was maintained until the end of the experiment. They were kept in 60 L plastic seawater tanks for 14 days. During the acclimation period, water was replaced at approximately 50% daily, and the sea urchins were fed with kelp *Laminaria japonica* at 5% of their body weight per day. After acclimation, the healthy sea urchins were randomly divided into five groups of 15 individuals each and placed in 60 L plastic tanks in preparation for the subsequent experiments.

### 2.2. Strain Isolation and Identification

The isolation method was performed as described [3]. We selected 10 sea urchins showing apparent red spots, which were raised in the laboratory at Dalian Ocean University. The sea urchins were rinsed with UV-sterilised seawater. The peristomal membrane of diseased *S. intermedius* was cut open using sterile scissors. We used a pipette to aspirate 100 μL of body cavity fluid and streaked it onto 2% NaCl Nutrient Agar (NA) plates for isolation. The colonies were cultured at 28 °C for 24 h, and their colony morphology was observed. Colonies with consistent morphology were selected and streaked for further isolation and identification. This procedure was repeated 3–4 times until a pure culture of the dominant strain was obtained. The identified strain was preserved in glycerol and stored at −80 °C for further use.

The identified strains were submitted to Biotechnology Bioengineering (Shanghai) Ltd. (Shanghai, China) for sequencing analysis. The bacterial genomic DNA was extracted using a bacterial genomic DNA extraction kit (Tiangen, Beijing, China) according to the manufacturer’s instructions. Two universal bacteria primers [27] for the 16S rDNA gene were synthesized by Biotechnology Bioengineering (Shanghai) Ltd. (Table S1). The quantitative qRT-PCR reaction mixture consisted of a total volume of 20 µL, comprising 2 µL of cDNA template, 10 µL of 2× SYBR Green Master Mix (TaKaRa, Shiga, Japan), 0.8 µL of each primer, and 6.4 µL of PCR-grade water. The PCR conditions were set as follows: initial denaturation at 94 °C for 5 min, denaturation at 94 °C for 45 s, annealing at 55 °C for 45 s, extension at 72 °C for 90 s, followed by 30 cycles, and final extension at 72 °C for 10 min. Finally, the obtained sequences were subjected to homology searches using GenBank, and a phylogenetic tree was built based on previous studies [28].

### 2.3. Drug Sensitivity Test of Isolated Strains

The antibiotic resistance and susceptibility of the target strain (HZ-3-2) to 18 commonly used antibiotics were determined using the Kirby–Bauer disc diffusion method. A 100 µL aliquot of bacterial suspension (The concentration is 10^7^ CFU/mL) culture was evenly spread on 2% NaCl NA. After the bacterial suspension was fully absorbed, the plate was divided into four quadrants and a circular antibiotic disc was placed in each quadrant. The plate was then incubated at 28 °C for 24 h. The diameter of the inhibitory zone was measured by using vernier calipers. Finally, we determine the sensitivity of the isolated strains to various antibiotics, in accordance with the specified sensitivity ranges.

### 2.4. Experimental Infection on V. splendidus

The experimental infection method was performed as described previously [1]. The isolated bacterial suspension was cultured on agar plates containing 2% NaCl and incubated at 27 °C for 24 h in a biochemical incubator. Single colonies were then selected and inoculated into a 2% NaCl NA liquid medium. The culture was incubated overnight at 27 °C in a shaking incubator, followed by centrifugation at 3000 rpm for 10 min. After centrifugation, the supernatant was discarded. Later, a 1000 μL aliquot of sterilized seawater was added to the centrifuge tube, and the pellet was transferred into it. The cell concentration was determined using a hemocytometer. Three bacterial suspensions were prepared at concentrations of 10^3^ CFU/mL, 10^5^ CFU/mL, and 10^7^ CFU/mL.

Five groups of sea urchins were selected, four of which were wounded by scraping 1.5 cm^2^ of spines. Three of the scraping spine groups were randomly selected and immersed in 3 L of bacterial suspension at concentrations of 10^3^ CFU/mL, 10^5^ CFU/mL, and 10^7^ CFU/mL for a duration of two hours, respectively. To prevent oxygen depletion in the small water volume, the infection procedure was repeated every two hours over three cycles, totalling six hours of infection. Sea urchins that were immersed in a bacterial suspension with a concentration of 10^7^ CFU/m were designated group M. The remaining group of sea urchins was immersed directly in seawater to serve as the control group (designated as group C). Additionally, the group that did not undergo spine scraping was directly immersed in a bacterial suspension at 10^7^ CFU/mL. After completing the above procedures, the sea urchins were transferred to a water tank, and daily observations along with clinical signs were documented. The test was conducted over seven days.

After the experimental infection, we dissected the sea urchins that survived in the bacterial suspension at 10^7^ CFU/mL. We used three samples, each composed of two randomly selected pieces of intestine, for transcriptome sequencing, which comprised three pooled replicates (n = 3).

### 2.5. Whole-Genome Sequencing and Analysis

DNA was extracted using MagPure Bacterial DNA Kit (D6361-02, Magen, Foshan, China). The concentration of DNA was determined using the Qubit 4.0 (Thermo, Waltham, MA, USA, Q33226). And the integrity of the DNA was evaluated through 1% agarose gel electrophoresis.

The whole genome DNA was randomly fragmented to an average size of 200–400 bp. The fragments were obtained through end-repair, 3′ adenylation, adapter ligation, and PCR amplification. After purification with magnetic beads, the Qubit 4.0 fluorometer was used to validate the library, while the library length was evaluated using 2% agarose gel electrophoresis. The qualified libraries were sequenced using the Illumina NovaSeq 6000 platform at Sangon Biotech (Shanghai, China). Following the sequencing process, raw reads were filtered utilizing Trimmomatic (v0.36) to eliminate adaptors and low-quality reads, resulting in the clean reads. Genome assembly was conducted employing SPAdes (v3.15), and Gapfiller (v1.11) was used for the purpose of filling gaps. We used Pilon (v3.5.0) to improve the accuracy of draft genomes by correcting base errors and filling gaps. Gene prediction was performed using Prokka (v1.1). Tandemly repeated DNA motifs were identified with TRF (v4.09). Finally, we utilized Circos software (v0.66) to create circular genome maps.

### 2.6. Genome Functional Annotation

Gene predictions and annotations were generated using Prokka (Version 1.10) and the National Center for Biotechnology Information (NCBI) non-redundant protein (NR) database [29]. Subsequently, Cluster of Orthologous Groups (COG) [30], Gene Ontology (GO) [31], and the Kyoto Encyclopedia of Genes and Genomes (KEGG) [32] were used to predict gene functions. The Comprehensive Antibiotic Resistance Database (CARD) [33] was consulted to predict which were the virulence genes and antibiotic resistance genes.

### 2.7. Libraries Construction and High-Throughput Sequencing

Total RNA was extracted from the tissues using TRIzol reagent (Invitrogen, Carlsbad, CA, USA) according to the manufacturer’s protocol [34]. Subsequently, total RNA was assessed for quality and quantity using an Agilent 2100 Bioanalyzer (Agilent, Santa Clara, CA, USA). The sample standards were OD260/280 ≥ 1.8.

Library preparation was performed using the Optimal Dual-mode mRNA Library Prep Kit (BGI, Shenzhen, China). A defined amount of RNA was denatured at an appropriate temperature to disrupt its secondary structure, and the mRNA was subsequently enriched using oligo (dT)- attached magnetic beads. RNA was fragmented using fragmentation reagents after incubating at an appropriate temperature for a specified period.

First-strand cDNA was generated using random hexamer-primed reverse transcription, followed by second-strand cDNA synthesis. The synthesized double-strand cDNA is subject to an end-repair reaction. After the end-repair of the cDNA, a single ‘A’ nucleotide was added to the 3′ ends of the blunt fragments through an A-tailing reaction. Subsequently, an adaptor-ligation reaction system was configured to ligate adaptors to the cDNA, and the library products were amplified PCR and subjected to quality assessment.

Next, the single-stranded library products were generated through denaturation. A circularisation reaction system was established to create the single-stranded cyclized DNA products. Any uncyclised single-stranded linear DNA molecules were digested. The final single-stranded circularised library was amplified using phi29 and rolling circle amplification (RCA) to produce DNA nanoballs (DNBs), each containing more than 300 copies of the initial single-stranded circularised library molecule. The DNBs are loaded into the patterned nanoarray, and PE 100/150 base reads are generated on the G400/T7/T10 platform (BGI-Shenzhen, Shenzhen, China).

### 2.8. Transcriptome Assembly and Annotation

The raw data was filtered with SOAPnuke (v1.6.5) [35] by removing reads containing adapters (adapter trimming). Clean reads were obtained and stored in FASTQ format. The clean data were mapped to the reference genome by HISAT (v2.2.1) [36]. The clean data were mapped to the assembled unique gene by Bowtie2 (v2.4.5) [37].

The expression level of genes was calculated by RSEM (v1.3.1) [38]. Transcripts were reconstructed using StringTie (v2.2.1) [39], and cuffmerge was employed to integrate the assembled transcripts. Cuffcompare was employed to compare the integrated transcripts with a reference annotation. Transcripts with class code types u, i, o, and j were selected as novel transcripts. The protein-coding potential of the novel transcripts was predicted using CPC (v0.1). Gene annotation was performed using public databases (e.g., KEGG, GO).

Differential gene analysis was conducted between groups using DEGSeq. (Bioconductor version v3.20), with a fold change ≥ 2 and an Adjusted *p*-value ≤ 0.001. PoissonDis was used for between-sample differential gene analysis, with a fold change of ≥2 and a false discovery rate (FDR) of ≤0.001. DEGs were functionally classified according to KEGG annotation results and official classification. KEGG enrichment analysis was performed using the phyper function in R software (v4.4.3). With *Q*-value ≤ 0.05 as the threshold, candidate genes that met this condition were defined as being significantly enriched.

### 2.9. Real-Time Fluorescent PCR (qRT-PCR) Gene Validation

To validate the RNA-seq results, six DEGs were selected for qRT-PCR (Table S2). Total RNA was extracted, and reverse transcription was performed as described previously [40]. The relative quantities of the target genes were calculated using *β-actin* from *S. intermedius* as an endogenous control gene. The qRT-PCR was performed using the LightCycler96 real-time system (Roche, Basel, Switzerland). The reactions were carried out according to the manufacturer’s instructions for FastStart Essential DNA Green Master (Roche, Switzerland). PCR primers were designed and synthesized by Sangon Biotechnology (Shanghai, China). The quantitative qRT-PCR reaction mixture consisted of a total volume of 20 µL, comprising 2 µL of cDNA template, 10 µL of 2 × SYBR Green Master Mix (TaKaRa, Tokyo, Japan), 0.8 µL of each primer, and 6.4 µL of PCR-grade water. The thermal cycling conditions were set: an initial denaturation at 95 °C for 30 s, followed by 45 cycles. Annealing and elongation phases were conducted at 95 °C for 5 s and 60 °C for 32 s. Three independent biological replicates and three technical replicates were performed for each group. Melt curve analysis of the amplification products confirmed the presence of a single PCR product at the end of the PCR reaction. The relative expression levels of target genes were calculated using the 2^−ΔΔCT^ method described previously [41].

## 3. Results

### 3.1. Pathogenic Bacterium of Sea Urchin Spotting Disease

In order to determine the strain of HZ-3-2, which was isolated from the outbreak of diseased *S. intermedius*, we conducted morphological observations, 16S rDNA, and whole-genome sequencing analysis. After 24 h of isolation and culturing, yellow, round colonies with smooth surfaces and regular edges were observed.

**Figure 1:**
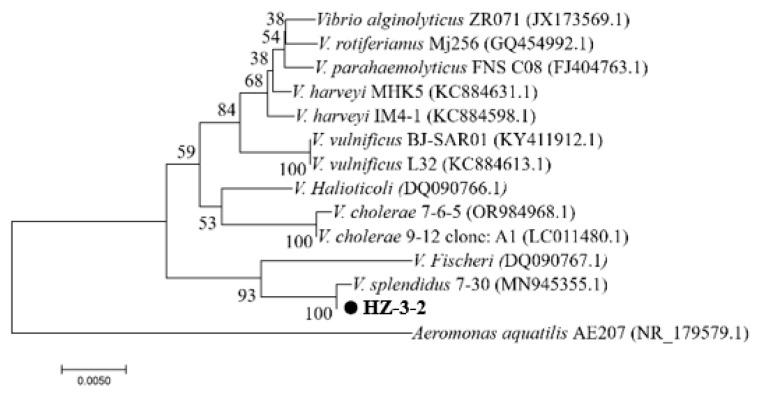
Phylogenetic tree of *Vibrio* spp. based on the 16S rDNA gene sequence. Note: Numbers in parentheses represent the sequences’ accession numbers in GenBank.

**Figure 2:**
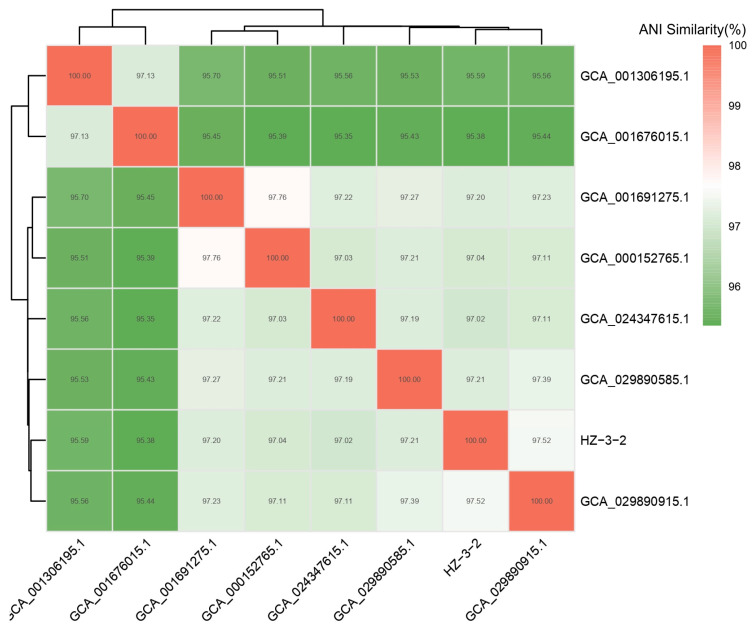
Average nucleotide identity distance heat map.

PCR amplification of 16S rDNA resulted in a 1210-bp fragment. We first sequenced the PCR fragment and then performed a homology search using GenBank. Based on the results, the strain was identified as *V. splendidus* with 99% similarity and an e-value of 0.0. Comparison results and PCR pictures can be found in Supplement S1 and Figure S1. Phylogenetic trees were constructed using Mega 5.0 software. *V. splendidus* and other *Vibrio* 16S rDNA sequences were selected from GenBank. The 16S rDNA phylogenetic tree results showed that HZ-3-2 and *V. splendidus* (GenBank accession No. MN945355.1) were clustered into one branch (Figure 1). The results of the Average Nucleotide Identity (ANI) analysis indicated that strain HZ-3-2 shares a 97.48% identity with *V. splendidus* S 27 09 GCA 029890915.1 (Figure 2 and Table S3). Therefore, based on the above results, it was preliminarily concluded that the HZ-3-2 was *V. splendidus*, and the identification result is at the species level.

### 3.2. Results of Experimental Infection on V. splendidus

**Figure 3:**
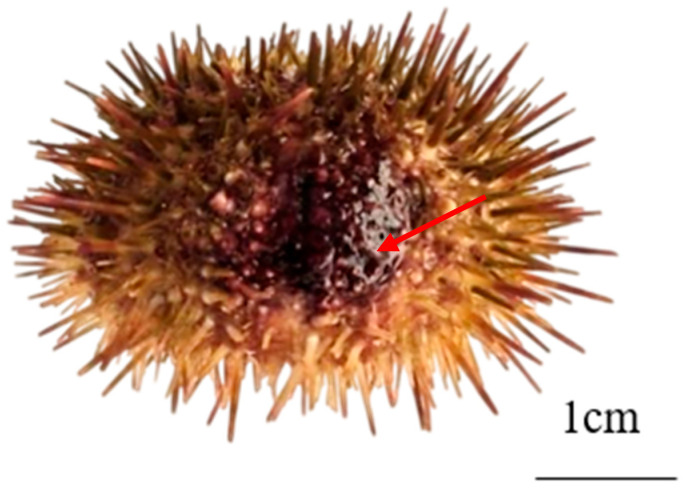
Signs of spotting disease in sea urchin. Note: The red arrows show signs of spotting disease.

We infected healthy urchins with HZ-3-2 to confirm that it was the pathogen responsible for spotting disease. Before the formal experiments, we conducted pre-experiments to determine that sea urchins died at a bacterial concentration of 10^5^ CFU/mL. The results showed that in all experimental groups, sea urchins displayed distinct red spots, a clinical sign of infection (Figure 3). During the experiment, the mortality rate increased with higher bacterial concentrations. In group M, with a bacterial concentration of 10^5^ CFU/mL, the average mortality rate reached 20% within 7 d. When the bacterial concentration was 10^7^ CFU/mL, the average mortality rate in group M was 66.7% (Table S4). The above analysis showed that *V. splendidus* is the pathogenic bacterium of spotting disease.

### 3.3. Whole-Genome Sequencing Analysis of HZ-3-2

#### 3.3.1. Genome Assembly

**Figure 4:**
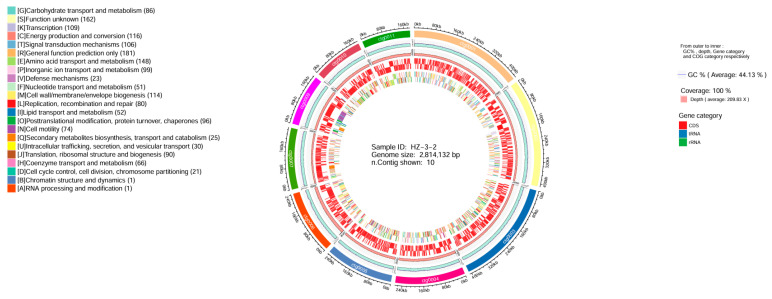
Genome circos diagram. Note: From the inside out, they are GC content, sequencing depth, gene element display, and COG function display, respectively.

Regarding the assembled genomic data, we used the Circos (v0.66) software to create a circular genome map, which included GC content, sequencing depth, Gene element content, and COG category. The results showed that the genome size was 2,814,132 bp, with a GC content of 44.13%, and a 100% coverage (Figure 4).

#### 3.3.2. NR Database Annotation

**Figure 5:**
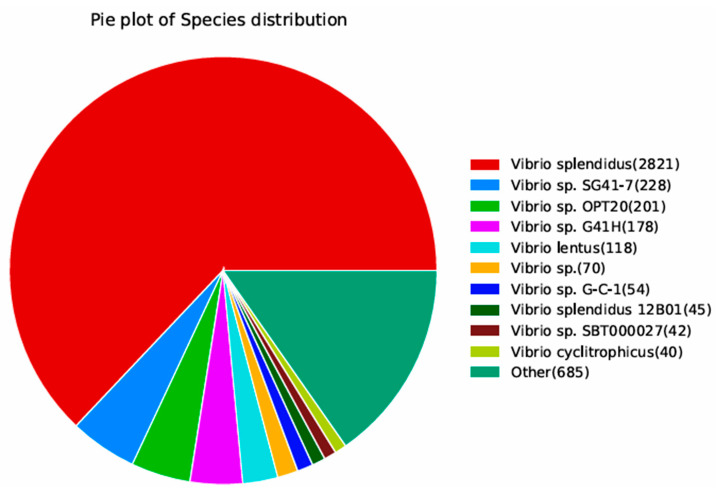
Species distribution map of the results of sequence alignment using the NR database.

We used the NR database annotation to reveal that *Vibrio* accounted for 84.72% of the organisms showing sequence homology with the HZ-3-2 strain. Among them, *V. splendidus* accounted for 62.94% and others accounted for 15.28% (Figure 5).

#### 3.3.3. Database Annotation

In the CARD database annotation analysis, we discovered that the HZ-3-2 strain contained CRP, rsmA, tet(35), ugd, catB9, and other resistance genes. These resistance genes produce resistance to fluoroquinolone antibiotics, macrolide antibiotics, penicillin, peptide antibiotics, phenicol antibiotics, etc. (Table S5).

The results of the COG, GO, and KEGG analyses of HZ-3-2 are presented in Figures S2, S3, and S4, respectively.

### 3.4. Results of Drug Susceptibility Testing of V. splendidus

To determine the susceptibility of *V. splendidus* to antibiotics, we chose cultured bacterial suspension for drug sensitivity testing. We analyzed the results based on drug sensitivity testing and the inhibition zone interpretation standard of the disk diffusion method. The results show that the *V. splendidus* strain is sensitive to several antibiotics, including cephalosporins (ceftazidime), tetracyclines (doxycycline), fluoroquinolones (enoxacin, levofloxacin, norfloxacin, and ciprofloxacin), sulfonamides (trimethoprim and sulfamethoxazole), aminoglycosides (gentamicin and streptomycin), polymyxin, and florfenicol. In addition, the *V. splendidus* strain showed intermediate sensitivity to other antibiotics, such as penicillin, Rifampin, Chloramphenicol, and erythromycin. Furthermore, the *V. splendidus* strain was resistant to vancomycin, Ofloxacin, and Tetracycline (Table 1).

### 3.5. Transcriptome Analysis of Sea Urchins Infected with V. splendidus in Different Ways

#### 3.5.1. Transcriptomic Data Quality Control Information

In this study, to investigate the molecular responses of sea urchins to infection with *V. splendidus*, we selected the intestines of sea urchins for transcriptome sequencing. And three biological replicates were set up for each group. The results yielded 202.56 million raw reads. After quality assessment, 199.05 million clean reads and 59.71 Gb of clean bases were obtained. The proportion of bases with a quality score ≥ Q20 and ≥Q30 (Ratio of bases with mass values > 20 and 30) for each sample exceeded 98% and 94%, respectively. The clean read rate was greater than 97%, demonstrating the high quality of the reads (Table S6).

#### 3.5.2. Statistical Analysis of Differentially Expressed Genes

**Figure 6:**
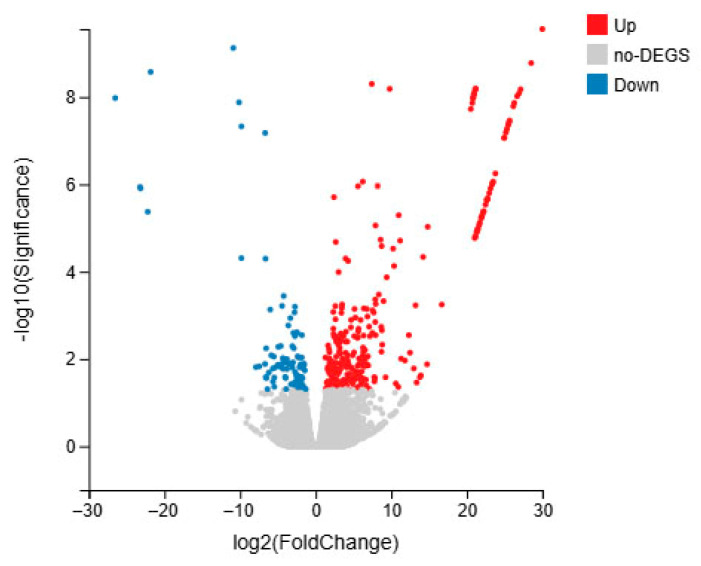
DEG expression changes in *S. intermedius* of immersed infection (group M vs. C).

We compared DEGs between the *S. intermedius* infected by different methods. Genes were selected using the thresholds |log2fc| ≥ 2 and *p* < 0.05, and the gene expression changes were visualized using a volcano plot. The comparison between groups M and C revealed 439 DEGs, with 245 genes upregulated and 194 genes downregulated (Figure 6).

#### 3.5.3. KEGG Pathway Classification Analysis

**Figure 7:**
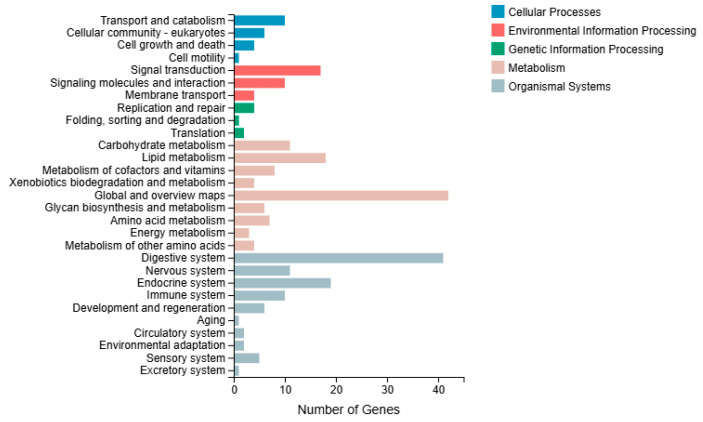
KEGG classification analysis in the immersed infection of *S. intermedius* (group M vs. C).

In the KEGG pathway classification analysis, the genes involved in KEGG metabolic pathways were categorized into five branches: cellular processes, environmental information processing, genetic information processing, metabolism, and organismal systems. Further classification and statistical analysis were conducted within each branch. The cellular processes category primarily involves transport, catabolism, and cellular community-eukaryotes. In the classification of environmental information processing, enrichment was predominantly in signal transduction. In the genetic information processing category, group M was primarily classified under replication and repair. In the organismal systems category, the digestive, endocrine, and immune systems were the most enriched (Figure 7).

#### 3.5.4. KEGG Pathway Enrichment Analysis

**Figure 8:**
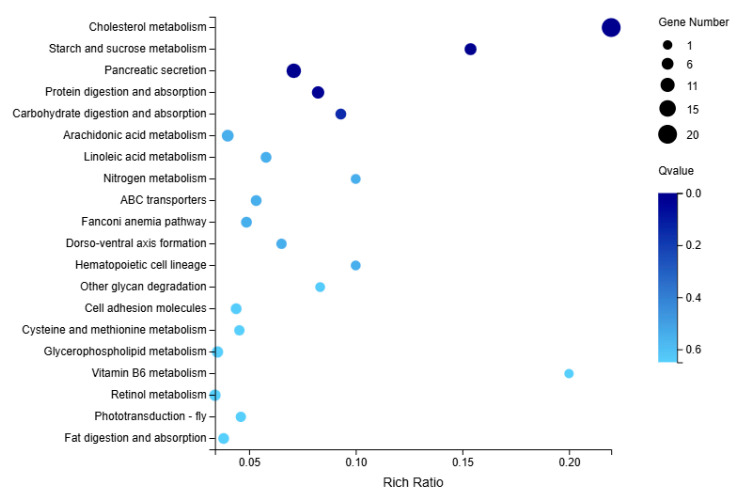
KEGG enrichment analysis in the immersed infection of *S. intermedius* (group M vs. C).

The top 20 most significantly correlated pathways were selected for KEGG enrichment analysis, with 158 DEGs enriched in group M. Compared to group C, group M was primarily enriched in cholesterol metabolism and starch and sucrose metabolism (Figure 8).

### 3.6. Transcriptome Quality Analysis Based on qRT-PCR Verification

**Figure 9:**
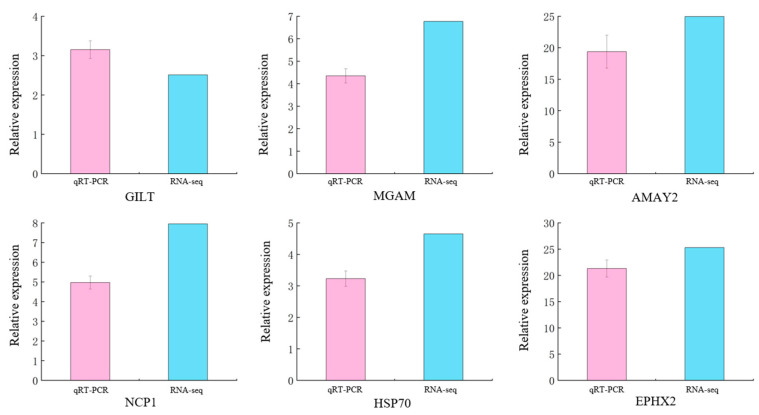
Verification of DEGs using qRT-PCR in immersed infected ways of *S. intermedius*. Note: The type of error bars is mean ± SD.

In order to determine the accuracy of the DEGs identified in the RNA-seq expression analysis, we selected 6 DEGs from the mRNA library for verification. These genes may be involved in sea urchins’ energy metabolism, immunity, and cholesterol secretion. The qRT-PCR results were significantly correlated with the RNA-seq results (*p* < 0.05). We find that qRT-PCR and RNA-seq show the same trend. Therefore, it was confirmed that the transcriptome data of the *S. intermedius* used in this study are reliable (Figure 9).

## 4. Discussion

### 4.1. V. splendidus Is the Pathogen of S. intermedius Spotting Disease

With the expansion of the sea urchin aquaculture industry, bacterial infections remain a major challenge, and spotting disease is one of the most severe threats [3]. The 16S rDNA gene sequence is the most widely employed genetic marker for bacterial taxonomy [42]. However, due to the exceptionally high similarity of 16S rDNA gene sequences among Vibrio species, the precision of species-level identification is constrained [43]. Therefore, we performed 16S rDNA and whole-genome sequencing of the isolated bacterial strain, confirming its identification as *V. splendidus* at the species level. The experimental results indicated that clinical signs of artificially infected *S. intermedius* were similar to those of naturally infected sea urchins, and the strains isolated from the diseased sea urchins were the same type. In this experiment, *S. intermedius* suffering from spotting disease exhibited red spots on the body wall, spine loss, reduced vitality, and a noticeable decline in tube foot adhesion, which is similar to findings of previous research [25,44]. *V. splendidus* is a Gram-negative bacterium belonging to the family *Vibrionaceae*, genus *Vibrio*, and is widely distributed in marine environments [45]. Different strains of *V. splendidus* have been linked to the mortality of Atlantic cod (*Gadus morhua*) [46], turbot larvae (*Scophthalmus maximus*) [45], and oysters (*Crassostrea gigas*) [47]. They are also associated with ulcerative syndrome in sea cucumbers (*Apostichopus japonicus*) [48], which seriously threatens the aquaculture industry’s growth.

Infections in marine systems are usually characterized by complex interactions among multiple microbial species, some of which may be pathogenic, while others are opportunistic [49,50]. Previous studies have found that spotting disease can be caused by several bacteria [12,14,15,16]. However, the underlying mechanism of infections related to the spotting disease remains unknown and requires further study.

### 4.2. Spotting Disease Prevention and Treatment Recommendations

This study utilised varying concentrations of *V. splendidus* to infect *S. intermedius*, finding that higher bacterial concentrations led to a gradual increase in sea urchin mortality. These results are consistent with prior research findings [44]. In immersed experiments, the disease did not occur without damage to the body surface. Therefore, we suggest that damage to the body surface of sea urchins may be an important factor in causing spotting disease. In sea urchin aquaculture, controlling stocking density (the current consensus is 33.7 g/m^2^ to 2.7 kg/m^2^ [51,52]) is essential to reduce test damage that may allow sea urchins to avoid *V. splendidus* infection. At the same time, we can reduce the rate of disease and deaths by using segregation methods in sea urchin aquaculture [53,54].

It is widely recognised that the reasonable use of antibiotics is an effective strategy for preventing and controlling diseases in aquatic species. [23]. The antimicrobial susceptibility assays in this study revealed that *V. splendidus* is sensitive to norfloxacin, ofloxacin, enrofloxacin, and florfenicol. According to the guidelines from the Ministry of Agriculture and Rural Affairs of the People’s Republic of China on the regulated use of antibiotics in aquaculture [55], norfloxacin, and ofloxacin [56] are prohibited in aquaculture. Therefore, florfenicol and enrofloxacin are recommended as potential treatments for spotting disease induced by *V. splendidus*. However, the HZ-3-2 strain is highly sensitive to fluoroquinolone antibiotics and cephalosporins. This may be related to the weakening and loss of function caused by mutations or methylation in resistance genes [57,58]. In treating spotting disease, we can isolate and identify its pathogenic bacteria at first. After that, we can perform drug testing to screen for drugs sensitive to the causative microorganisms to prevent and control the spotting disease.

### 4.3. Molecular Responses of Sea Urchins Infected with V. splendidus

Prior research has demonstrated that *V. splendidus* profoundly threatens the survival of various marine species, thus hindering the growth of the aquaculture industry [47,59]. This study confirms *V. splendidus* is the pathogenic bacterium of spotting disease in *S. intermedius*. Nevertheless, the molecular responses of *S. intermedius* to infection by *V. splendidus* remain primarily uncharacterized, posing a challenge for effective prevention and control. In this study, we used the immersion method to simulate natural conditions, strengthening the validity of the experimental findings. We found that *S. intermedius* mounts a complex molecular response and involving numerous DEGs following the infection of *V. splendidus*. This information enhances our understanding of the molecular responses of sea urchin intestines to the infection of *V. splendidus*.

As invertebrates, sea urchins’ immune response primarily relies on coelomic fluid phagocytes to counter invading pathogens [25,60]. The intestine is recognized as an immune organ [26]. However, the kind of immune effects it exerts and its potential role in molecular responses after *V. splendidus* infection in sea urchins remain unknown.

The experimental results indicate that the starch and sucrose metabolism pathway was significantly activated in group M, with AMY2A and MGAM expression being upregulated. *AMY2A* [61,62] and *MGAM* [63,64] are crucial in the metabolic processing of starch and carbohydrates. The upregulation of both genes promotes the absorption of carbohydrates. This phenomenon may indicate a correlation between the energy metabolism and bacterial infection in sea urchins.

The cholesterol metabolism pathway showed the most significant response in group M, with *NPC1* expression significantly upregulated. *NPC1* and *NPC2* have a synergistic effect that enhances the efficiency of cholesterol binding and transport [65,66]. These findings suggest a noticeable increase in cholesterol expression within the intestine of *S. intermedius* after the infection of *V. splendidus*. Cholesterol possesses pro-inflammatory properties that promote macrophage accumulation, trigger inflammatory responses, and induce apoptosis, with excessive levels potentially affecting immune regulation [67]. That likely reflects toxic damage to the sea urchin following the bacteria’s invasion of its intestinal tissues. This discovery contributes to our understanding of the molecular response changes in *S. intermedius* infection with *V. splendidus*.

Moreover, *NPC1* is an essential antigen receptor and an important component of filovirus entry into the host to initiate infection and pathogenesis [68]. Whether *NPC1* also functions as a carrier or mediator for *V. splendidus* entry into the host is uncertain. That deserves further investigation in future studies.

## 5. Conclusions

This study confirmed *V. splendidus* as a causative agent of spotting disease in sea urchins. Based on antimicrobial susceptibility testing, florfenicol and enrofloxacin are potential treatments for *V. splendidus*-induced spotting disease. We used immersed methods to infect *S. intermedius* with *V. splendidus* and compared transcriptome sequencing results with the control group, ultimately identifying 439 DEGs. *S. intermedius* exhibits altered energy metabolism, cholesterol synthesis, and immune responses following *V. splendidus* infection, with starch and sucrose metabolism, and cholesterol metabolism pathways activated in the intestine. This transcriptome sequencing study provides valuable insights into the intestinal molecular response of *S. intermedius* to *V. splendidus* infection.

## Figures and Tables

**Table 1:** Drug sensitivity test result of HZ-3-2.

Type	Concentration μg/Disk	Inhibition Zone Standard Value			Diameter of the Inhibition Zone/mm
		R	I	S	
Ceftazidime	30	≤14	15–17	≥18	27.36 (S)
Ciprofloxacin	5	≤15	16–20	≥21	22.07 (S)
Doxycycline	30	≤12	13–15	≥16	23.82 (S)
Enoxacin	5	≤15	16–20	≥21	26.91 (S)
Erythromycin	15	≤13	14–22	≥23	14.50 (I)
Florfenicol	30	≤12	13–17	≥18	28.60 (S)
Ofloxacin	5	≤12	13–15	≥16	11.63 (R)
Polymyxin	300	≤8	8–11	≥12	19.13 (S)
Rifampin	5	≤16	17–19	≥20	19.97 (I)
Streptomycin	10	≤11	12–14	≥15	20.93 (S)
Chloramphenicol	30	≤12	13–17	≥18	15.30 (I)
Gentamicin	10	≤12	13–14	≥15	27.52 (S)
Levofloxacin	5	≤13	14–16	≥17	28.83 (S)
Norfloxacin	10	≤12	13–16	≥17	21.36 (S)
Penicillin	10	≤19	20–27	≥28	23.77 (I)
Tetracycline	30	≤14	15–18	≥19	13.85 (R)
TMP-SMX	1.25	≤10	11–15	≥16	23.33 (S)
Vancomycin	30	≤14	15–16	≥17	—

Note: “S” means sensitive; “I” means intermediate sensitivity; “R” means resistant.

## Data Availability

The original contributions presented in this study are included in the article, and all sequence data have been submitted to the NCBI Short-Read Archive (SRA) with the accession number PRJNA1202030. Further inquiries can be directed to the corresponding author.

## Supplementary Materials

The following supporting information can be downloaded at: https://www.mdpi.com/article/10.3390/microorganisms13092019/s1, Figure S1. PCR photo; Figure S2. Function classification statistics of COG functional genes. Figure S3. GO function annotation classification statistics chart. Figure S4. KEGG annotation classification statistics. Table S1. List of primers used for 16S rDNA validation. Table S2. List of primers used for qRT-PCR validation. Table S3. Candidate species with the most optimal Average Nucleotide Identity comparison outcomes. Table S4. Table of immersed infection results (group M). Table S5. CARD database annotation statistics. Table S6. Quality of the Transcriptome Sequencing Data. Supplement S1. 16S result.

## Abbreviations

DEGs	Differentially expressed genes
UV	Ultraviolet
NA	Nutrient Agar
PCR	Polymerase Chain Reaction
HGAP	Hierarchical Genome Assembly Process
COG	Cluster of Orthologous Groups
NR	non-redundant
KEGG	Kyoto Encyclopedia of Genes and Genomes
GO	Gene Ontology
CARD	Comprehensive Antibiotic Resistance Database
RCA	rolling circle amplification
DNABs	DNA nanoballs
PE	Paired-end
DEGSeq	Differentially Expressed Genes from RNA-seq data
FDR	false discovery rate
qRT-PCR	Real-Time Fluorescent PCR
Q20	Ratio of bases with mass values >20
Q30	Ratio of bases with mass values >30
