# Supplementary material for: Whole-Genome-Sequencing Analysis of the Pathogen Causing Spotting Disease and Molecular Response in the Strongylocentrotus intermedius
Source: Microorganisms. 2025 Aug 29;13(9):2019. doi: 10.3390/microorganisms13092019 (PMC12471893; doi:10.3390/microorganisms13092019)
Supplement: Supplementary file 1 [file microorganisms-13-02019-s001.zip › Figure S2. COG Database Annotation.pdf]

The COG annotation indicated that the most prevalent groups consisted of E (Amino acid transport and metabolism), K (Transcription), and R (General function prediction only). A (RNA processing and modification), B (chromatin structure and dynamics), W (extracellular structures), Y (nuclear structure), and Z (cytoskeleton) were the five categories with the lowest proportions (Figure. S2).

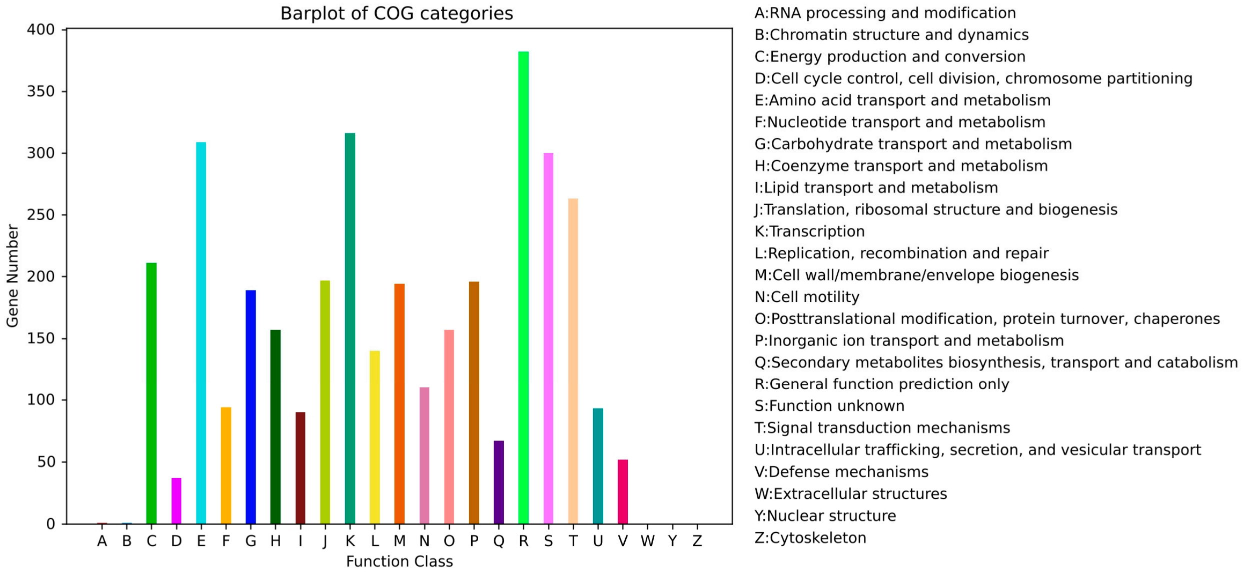

Figure S2. Function classification statistics of COG functional genes. Note: The abscissa indicates the content of each COG classification, while the ordinate indicates the relative abundance of the corresponding functional genes.
