# Supplementary material for: Whole-Genome-Sequencing Analysis of the Pathogen Causing Spotting Disease and Molecular Response in the Strongylocentrotus intermedius
Source: Microorganisms. 2025 Aug 29;13(9):2019. doi: 10.3390/microorganisms13092019 (PMC12471893; doi:10.3390/microorganisms13092019)
Supplement: Supplementary file 1 [file microorganisms-13-02019-s001.zip › Figure S3. GO Database Annotation.pdf]

In the GO database annotation analysis, genes are categorized into three main categories: biological process, cellular component, and molecular function. The HZ-3-2 strain had 1543 annotations pertaining to biological process, 235 annotations associated with cellular component, and 692 annotations related to molecular function. Among the 36 functional components, gene annotations mainly focused on cellular process, metabolic process, and catalytic activity (Figure S3).

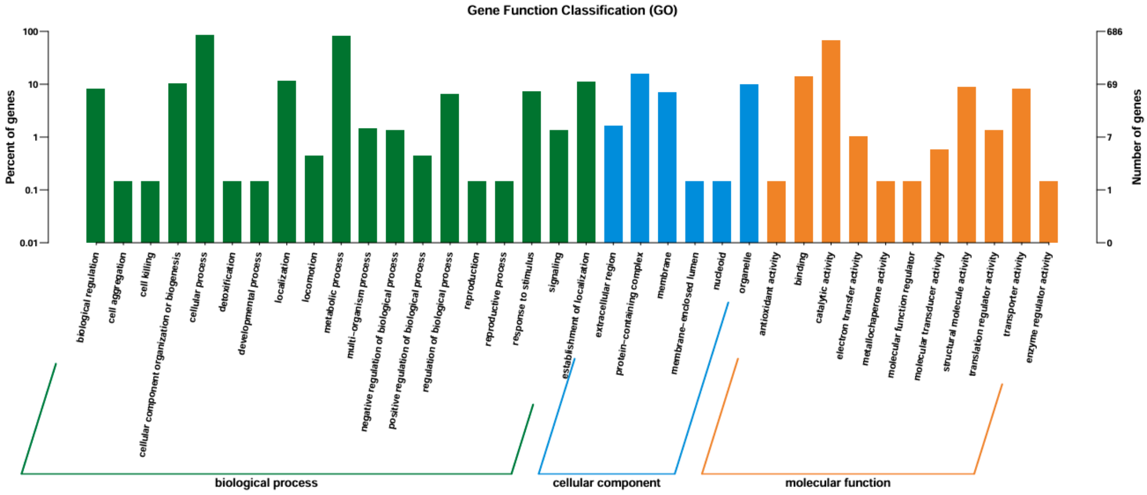

Figure S3. GO function annotation classification statistics chart. Note: The abscissa represents the content of each GO category, while the left side of the ordinate represents the percentage of genes, and the right side of the ordinate represents the number of genes.
