# Supplementary material for: Whole-Genome-Sequencing Analysis of the Pathogen Causing Spotting Disease and Molecular Response in the Strongylocentrotus intermedius
Source: Microorganisms. 2025 Aug 29;13(9):2019. doi: 10.3390/microorganisms13092019 (PMC12471893; doi:10.3390/microorganisms13092019)
Supplement: Supplementary file 1 [file microorganisms-13-02019-s001.zip › Figure S4. KEGG Database Annotation.pdf]

In the GO database annotation analysis, genes are categorized into five main categories: cellular processes, environmental information processing, genetic information processing, metabolism, and organismal systems. Among the 49 biological pathways, Membrane transport, Carbohydrate metabolism, and Overview were the three most highly represented in the pathways (Figure S4).

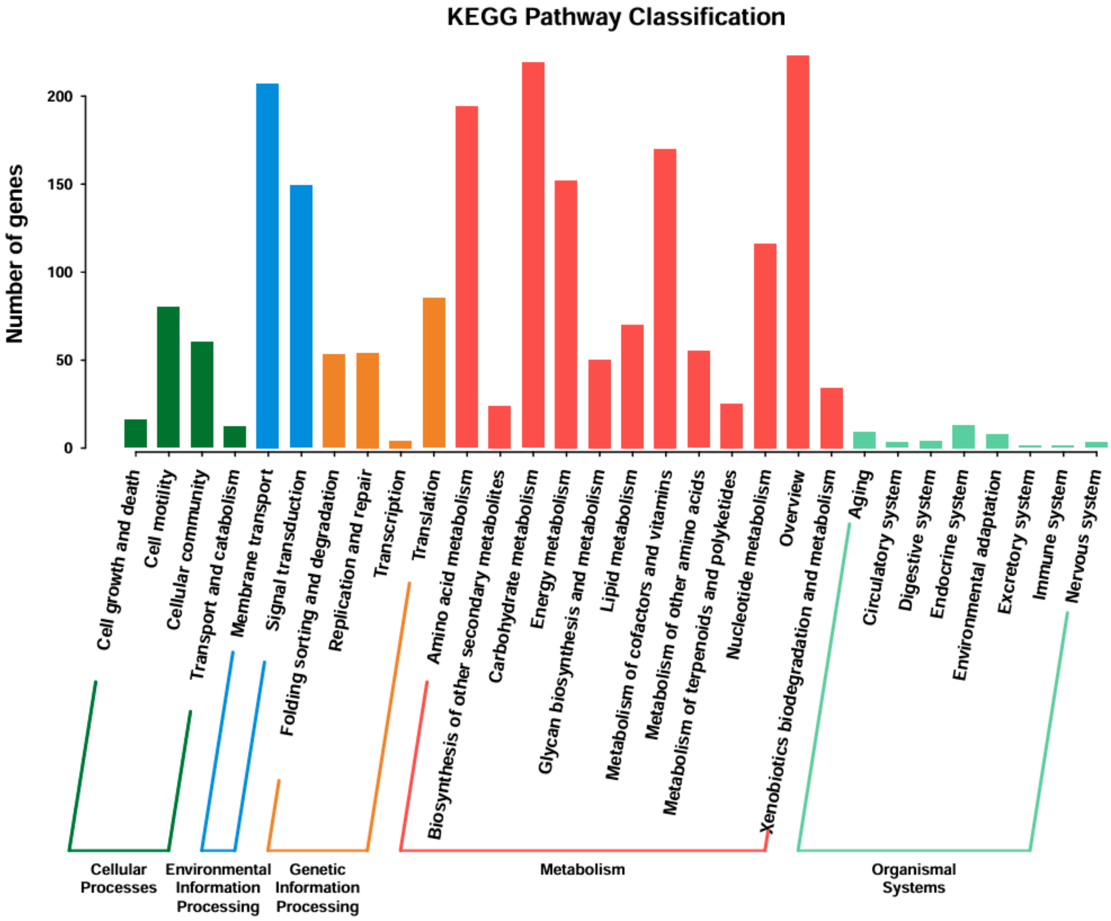

Figure S4. KEGG annotation classification statistics. Note: The abscissa represents the KEGG secondary classification, and the ordinate represents the number of genes.
