# Supplementary material for: Whole-Genome-Sequencing Analysis of the Pathogen Causing Spotting Disease and Molecular Response in the Strongylocentrotus intermedius
Source: Microorganisms. 2025 Aug 29;13(9):2019. doi: 10.3390/microorganisms13092019 (PMC12471893; doi:10.3390/microorganisms13092019)
Supplement: Supplementary file 1 [file microorganisms-13-02019-s001.zip › Table S1. List of primers used for 16S rDNA validation..pdf]

Table S1. List of primers used for 16S rDNA validation.

Primer sequence (5′ to 3′)
F: AGAGTTTGATCCTGGCTCAG
R: GGTTACCTTGTTACGACTT-3′
