# Supplementary material for: Whole-Genome-Sequencing Analysis of the Pathogen Causing Spotting Disease and Molecular Response in the Strongylocentrotus intermedius
Source: Microorganisms. 2025 Aug 29;13(9):2019. doi: 10.3390/microorganisms13092019 (PMC12471893; doi:10.3390/microorganisms13092019)
Supplement: Supplementary file 1 [file microorganisms-13-02019-s001.zip › Table S2. List of primers used for qRT-PCR validation..pdf]

Table S2. List of primers used for qRT-PCR validation.

Gene names	Primer sequence (5′ to 3′)
GILT-F	AATGTGTCGGAAACCTCGTC
GILT-R	CAGTCTTAAGGGCCATGGAA
MGAM-F	GACTACATGGACCGCAACCT
MGAM-R	CAGGCCACACTTTTCCGTAT
AMY2A-F	GCCTTTAGGGACATGGTTGA
AMY2A-R	CCACTACTGGATGGGCACTT
NPC1-F	GTCAGCAACGATGACGAAGA
NPC1-R	ATACCGCATGTGAGGGCTAC
HSP70-F	GGTCGAGAACTGCGTGTCCT
HSP70-R	CCGTCGCCAGTACCTCGAAT
EPHX2-F	TTTCCTACCTGCGCTCTCAT
EPHX2-R	GAAGTCCAGCCTCCTCACAG
β-actin-F	AGAGGCGTAGAGGGAAAGAC
β-actin-R	ACAGGGAAAAGATGGCACAGA
