# Supplementary material for: Whole-Genome-Sequencing Analysis of the Pathogen Causing Spotting Disease and Molecular Response in the Strongylocentrotus intermedius
Source: Microorganisms. 2025 Aug 29;13(9):2019. doi: 10.3390/microorganisms13092019 (PMC12471893; doi:10.3390/microorganisms13092019)
Supplement: Supplementary file 1 [file microorganisms-13-02019-s001.zip › Table S3. Candidate species with the most optimal Average Nucleotide Identity comparison outcomes.pdf]

Table S3. Candidate species with the most optimal Average Nucleotide Identity comparison outcomes.

Subject	Candidate species	ANI
GCA 029890915.1	*Vibrio splendidus* S 27 09 GCA 029890915.1	97.4814
GCA 029890585.1	*Vibrio splendidus* S 30 08 A GCA 029890585.1	97.1888
GCA 001691275.1	*Vibrio splendidus* 13B01 GCA 001691275.1	97.1205
GCA 000152765.1	*Vibrio splendidus* 12B01 GCA 000152765.1	97.0011
GCA 024347615.1	*Vibrio splendidus* LMG 19031 GCA 024347615.1	96.9492
GCA 001306195.1	*Vibrio splendidus* UCD-SED7 GCA 001306195.1	95.5033
GCA 001676015.1	*Vibrio splendidus* UCD-FRSSP16 15 GCA 001676015.1	95.3765
