# Supplementary material for: Whole-Genome-Sequencing Analysis of the Pathogen Causing Spotting Disease and Molecular Response in the Strongylocentrotus intermedius
Source: Microorganisms. 2025 Aug 29;13(9):2019. doi: 10.3390/microorganisms13092019 (PMC12471893; doi:10.3390/microorganisms13092019)
Supplement: Supplementary file 1 [file microorganisms-13-02019-s001.zip › Table S4. Table of immersed infection results. (group A).pdf]

Table S4. Table of immersed infection results (group M).

Group	Concentration /(cfu·mL^{-1})	Number/ individual (s)	Number of deaths/individual(s)							Number of diseased /individuals	Prevalence
			1d	2d	3d	4d	5d	6d	7d		
1	10^3	15	0	0	0	0	0	0	0	0	0%
2	10^5	15	0	0	0	1	0	1	1	3	20%
3	10^7	15	0	0	2	3	2	2	1	10	66.7%
