# Supplementary material for: Whole-Genome-Sequencing Analysis of the Pathogen Causing Spotting Disease and Molecular Response in the Strongylocentrotus intermedius
Source: Microorganisms. 2025 Aug 29;13(9):2019. doi: 10.3390/microorganisms13092019 (PMC12471893; doi:10.3390/microorganisms13092019)
Supplement: Supplementary file 1 [file microorganisms-13-02019-s001.zip › Table S5. CARD database annotation statistics.pdf]

ARO_Name	GeneID	CARD
CRP	ctg0015_02957	ARO:3000518
QnrS5	ctg0012_02610	ARO:3002794
ugd	ctg0006_01674	ARO:3003577
catB9	ctg0007_01782	ARO:3002681
cprR	ctg0001_00154	ARO:3005063
rsmA	ctg0025_04087	ARO:3005069
tet(35)	ctg0019_03421	ARO:3000481
msbA	ctg0017_03285	ARO:3003950
baeR	ctg0012_02517	ARO:3000828
leuO	ctg0021_03650	ARO:3003843
YajC	ctg0010_02199	ARO:3005040
H-NS	ctg0019_03419	ARO:3000676

Drug_Class
fluoroquinolone antibiotic;macrolide antibiotic;penam
fluoroquinolone antibiotic
peptide antibiotic
phenicol antibiotic
peptide antibiotic
diaminopyrimidine antibiotic;fluoroquinolone antibiotic;phenicol antibiotic
tetracycline antibiotic
nitroimidazole antibiotic
aminocoumarin antibiotic;aminoglycoside antibiotic
disinfecting agents and antiseptics;nucleoside antibiotic
cephalosporin;disinfecting agents and antiseptics;fluoroquinolone antibiotic;glycopeptide antibiotic;glycylcyclin
cephalosporin;cephamycin;fluoroquinolone antibiotic;macrolide antibiotic;penam;tetracycline antibiotic

Resistance_Mechanism
antibiotic efflux
antibiotic target protection
antibiotic target alteration
antibiotic inactivation
antibiotic efflux;antibiotic target alteration
antibiotic efflux
antibiotic efflux
antibiotic efflux
antibiotic efflux
antibiotic efflux
antibiotic efflux
antibiotic efflux
