# Supplementary material for: Whole-Genome-Sequencing Analysis of the Pathogen Causing Spotting Disease and Molecular Response in the Strongylocentrotus intermedius
Source: Microorganisms. 2025 Aug 29;13(9):2019. doi: 10.3390/microorganisms13092019 (PMC12471893; doi:10.3390/microorganisms13092019)
Supplement: Supplementary file 1 [file microorganisms-13-02019-s001.zip › Table S6. Quality of the Transcriptome Sequencing Data.pdf]

Table S6. Quality of the Transcriptome Sequencing Data

Commented [M1]: We revised it to Table S6, please confirm.

Groups	Sample	Total Raw Reads (M)	Total Clean Reads (M)	Total Clean Bases (Gb)	Clean Reads Q20 (%)	Clean Reads Q30 (%)	Clean Reads Ratio (%)
	a	22.56	22.08	6.62	98.96	94.83	97.87
M	b	22.56	22.13	6.64	98.91	94.50	98.09
	c	22.40	22.13	6.64	98.89	94.44	98.79
	d	22.40	22.10	6.63	98.81	93.94	98.66
N	e	22.40	22.14	6.64	98.86	94.21	98.84
	f	22.56	22.13	6.64	98.88	94.27	98.09
	g	22.72	22.13	6.64	99.00	94.94	97.40
C	h	22.40	22.06	6.62	98.96	94.75	98.48
	i	22.56	22.15	6.64	98.95	94.72	98.18

Note: “B” means injected infection; “A” means immersed infection; “C” means control group; “raw reads” means raw sequences from high-throughput sequencing; “clean reads” mean score after removing the low-quality data in raw reads; “clean bases” mean valid sequence after filtering; “Q20” and “Q30” mean ratio of bases with mass values >20 and 30.
