# Supplementary figures and images for: Whole-Genome-Sequencing Analysis of the Pathogen Causing Spotting Disease and Molecular Response in the Strongylocentrotus intermedius
Source: Microorganisms. 2025 Aug 29;13(9):2019. doi: 10.3390/microorganisms13092019 (PMC12471893; doi:10.3390/microorganisms13092019)

Figure S1. PCR photo.

100-3000 bp Ladder-K

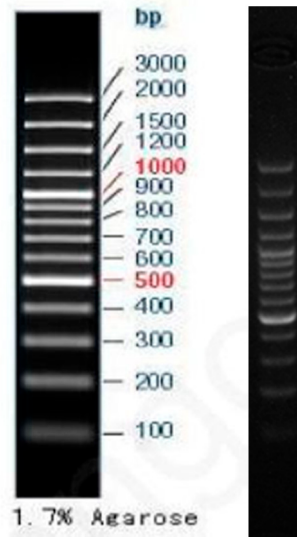

Supplement: Supplementary file 1 [file microorganisms-13-02019-s001.zip › Figure S1. PCR photo..pdf]
